# Supplementary figures and images for: Visual perception of texture regularity: Conjoint measurements and a wavelet response-distribution model
Source: PLoS Comput Biol. 2021 Oct 15;17(10):e1008802. doi: 10.1371/journal.pcbi.1008802 (PMC8550603; doi:10.1371/journal.pcbi.1008802)

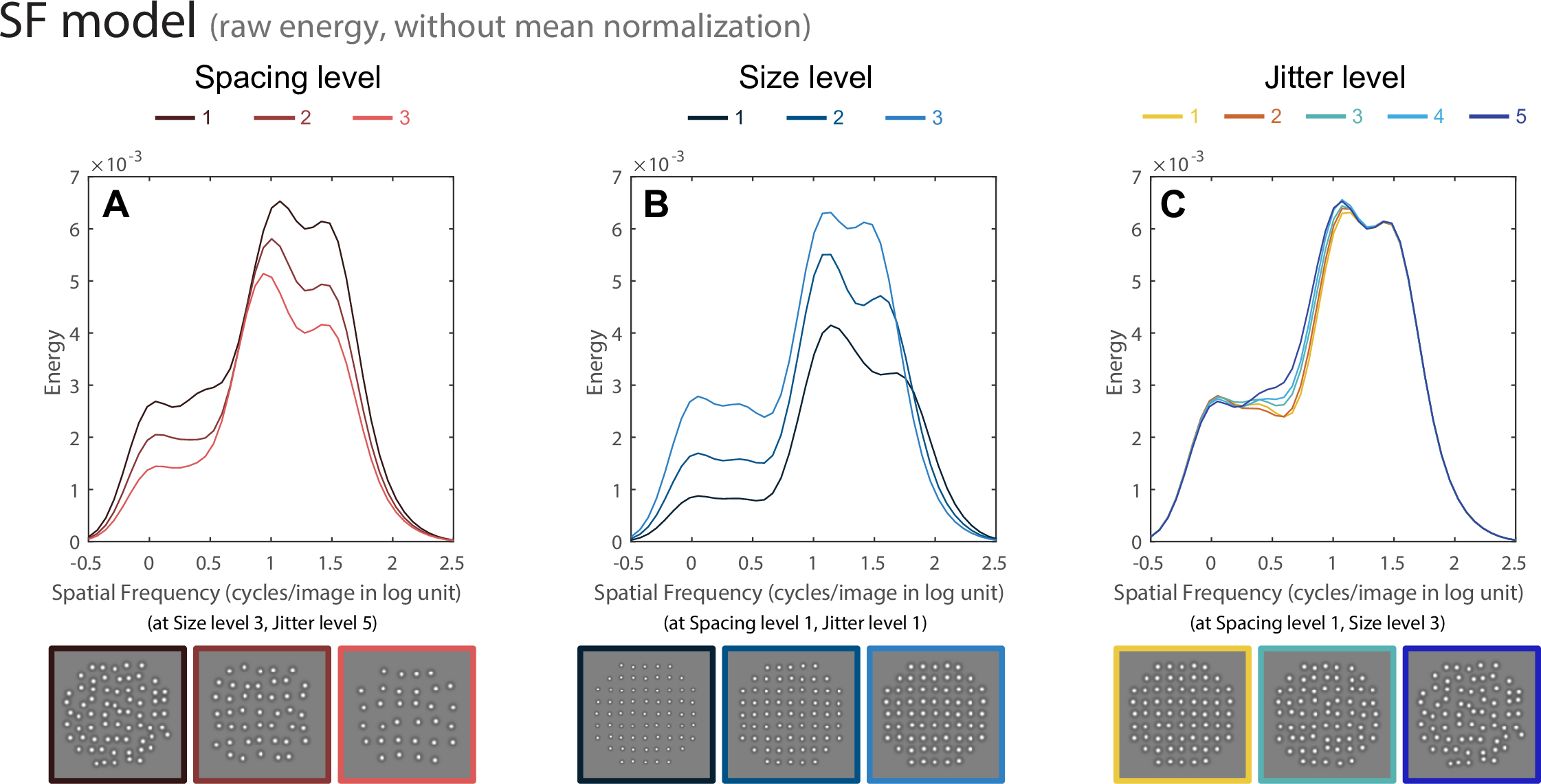

Supplement: S1 Fig — The same SF analysis as demonstrated in Fig 8 was used here but without mean normalization. See Fig 8 for details. (TIF) [file pcbi.1008802.s001.tif]

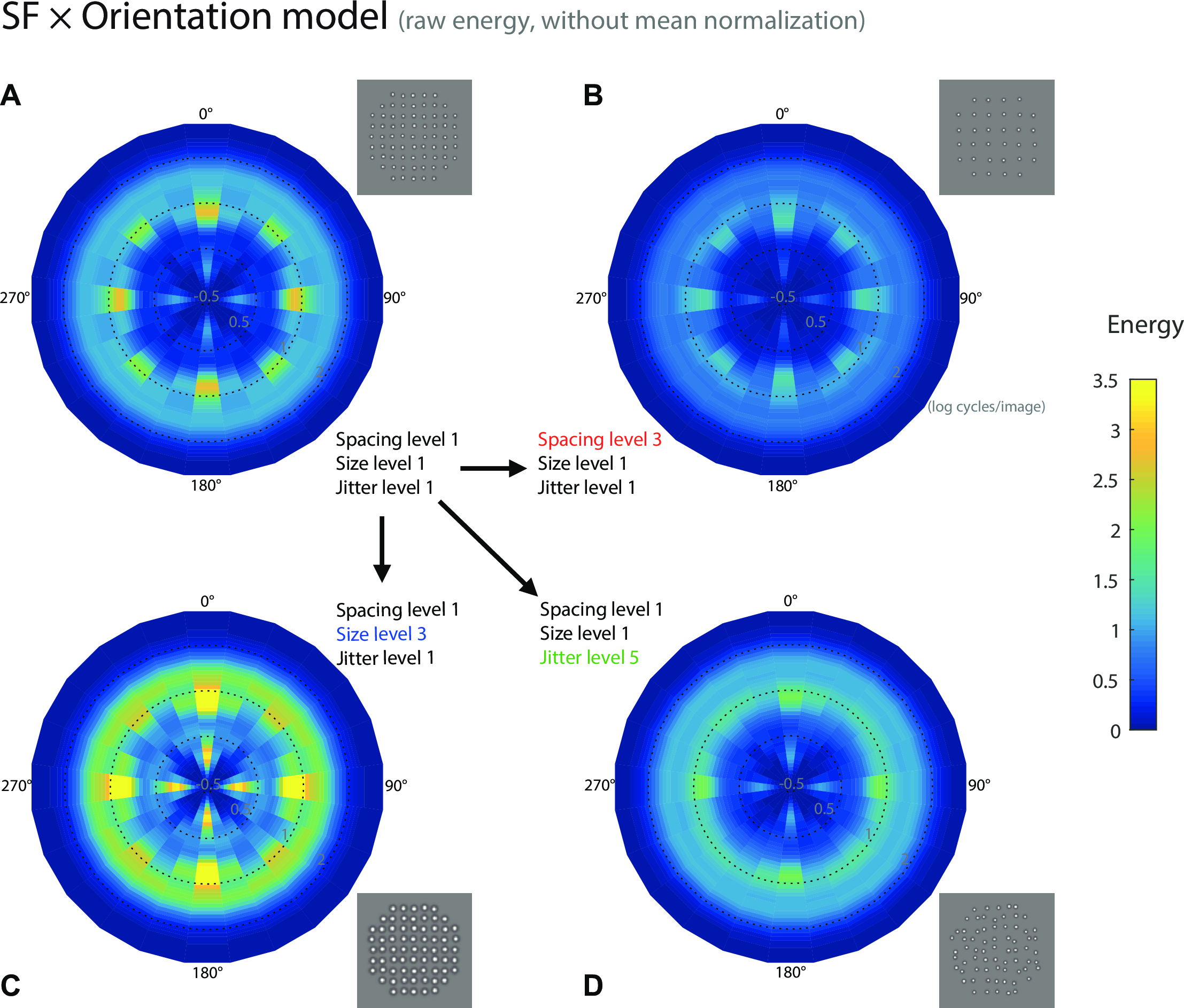

Supplement: S2 Fig — The same SF analysis as demonstrated in Fig 9 was performed but without mean normalization. See Fig 9 for details. (TIF) [file pcbi.1008802.s002.tif]

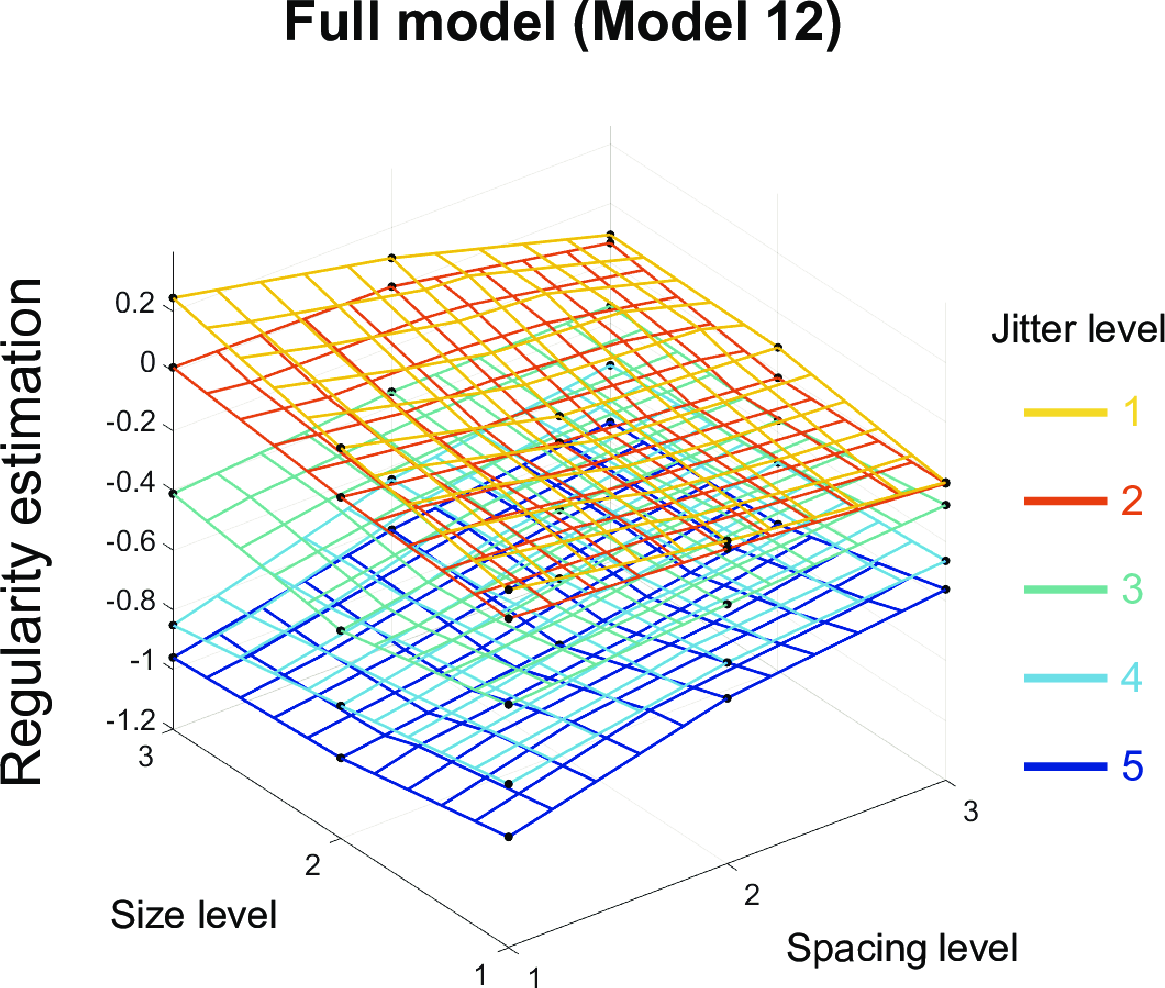

Supplement: S3 Fig — Color codes for the five jitter levels (yellow to blue). The image-computable models were used to predict the psychophysical results presented here. (TIF) [file pcbi.1008802.s003.tif]

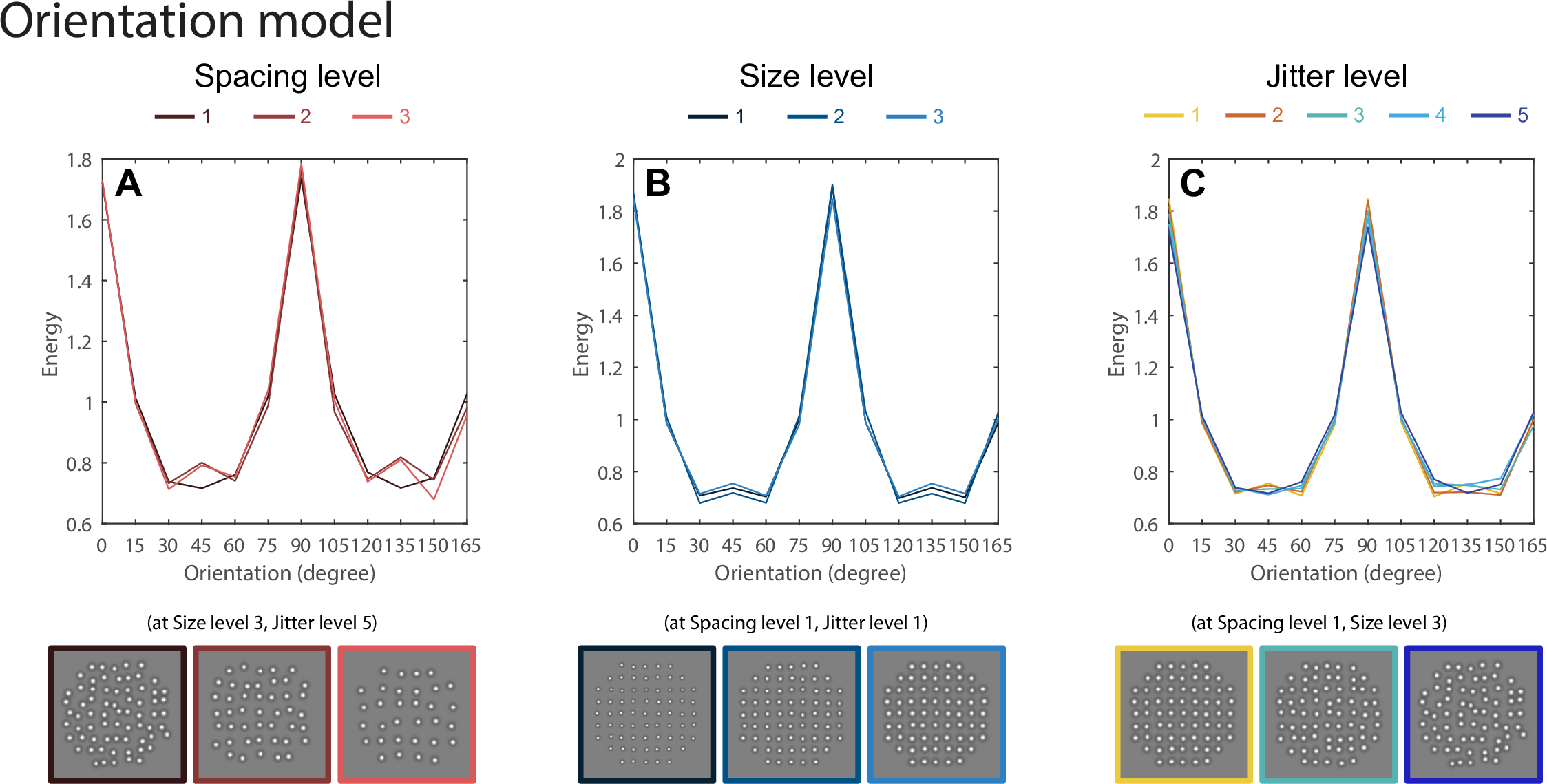

Supplement: S4 Fig — The same SF analysis as in Fig 8 was performed, except that the energy (ordinate) was normalized to equate total response across the 50 SF levels (Formula 3.2) and was plotted as a function of orientation (abscissa). Smaller element spacing (A), larger element size (B) and lower regularities (C) seem to make the orientation distribution slightly flatter. (TIF) [file pcbi.1008802.s004.tif]

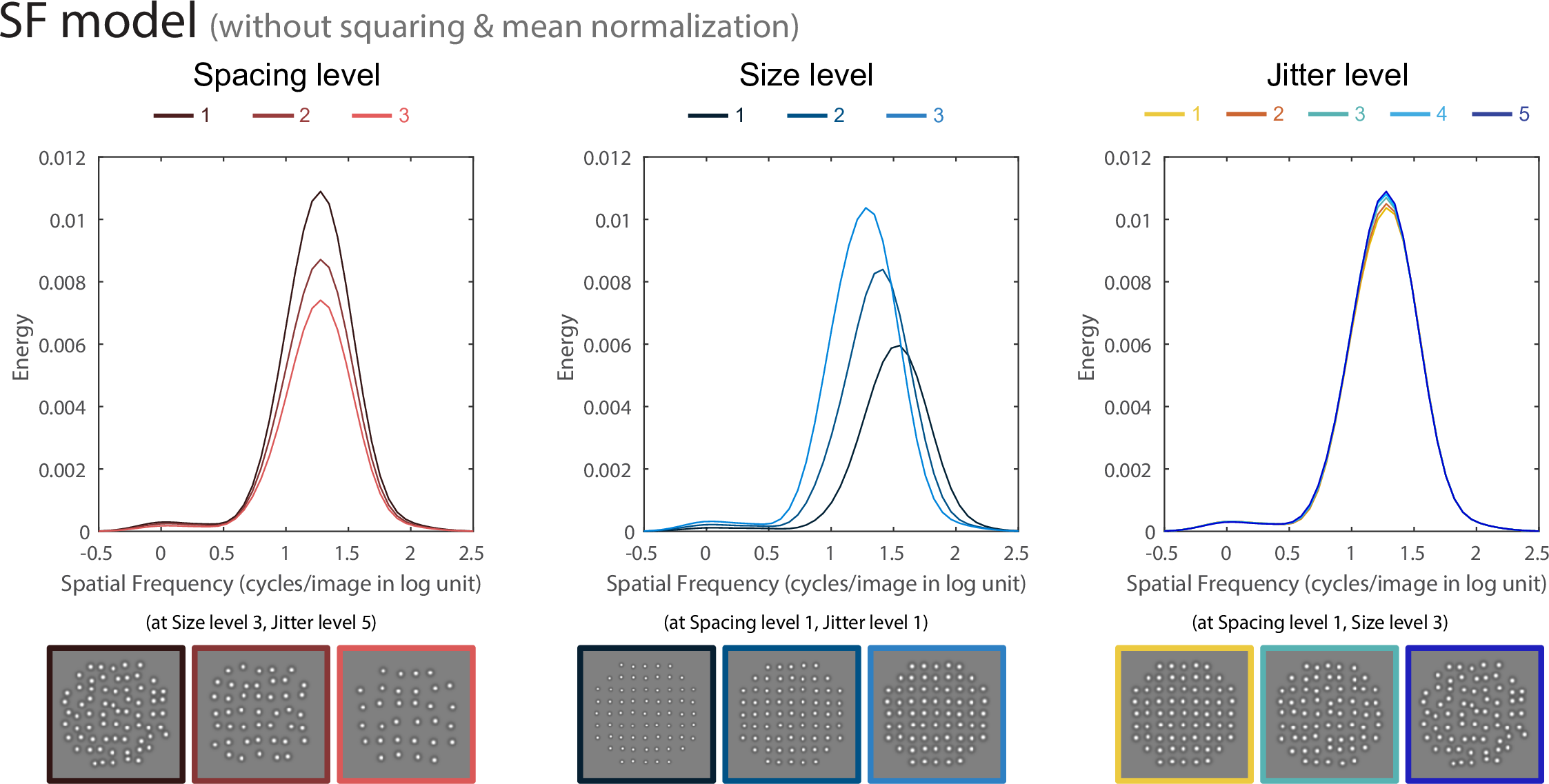

Supplement: S5 Fig — The same SF analysis as in S1 Fig was used, but without initial squaring of input images. (TIF) [file pcbi.1008802.s005.tif]

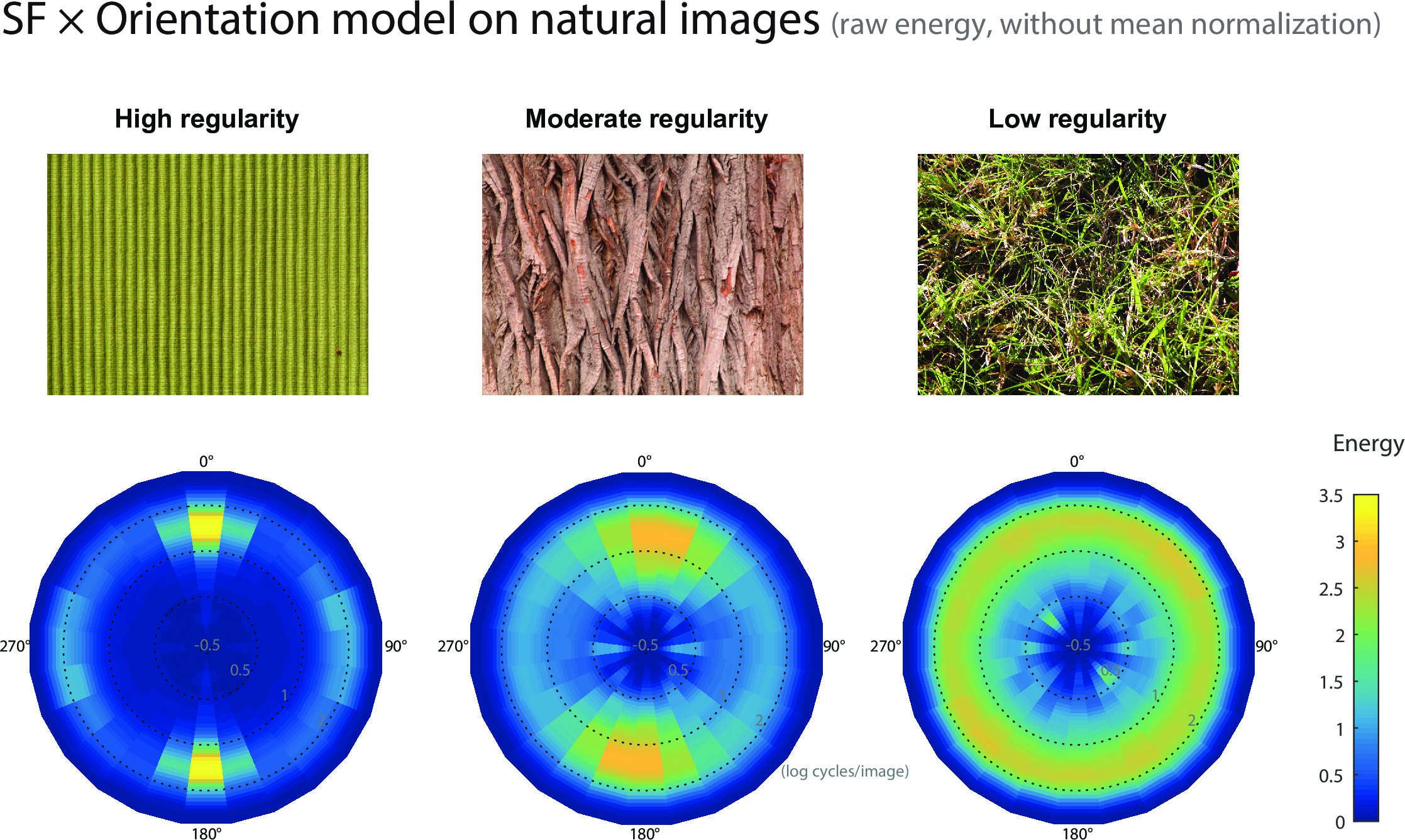

Supplement: S6 Fig — The same analysis as demonstrated in S2 Fig was performed. (TIF) [file pcbi.1008802.s006.tif]

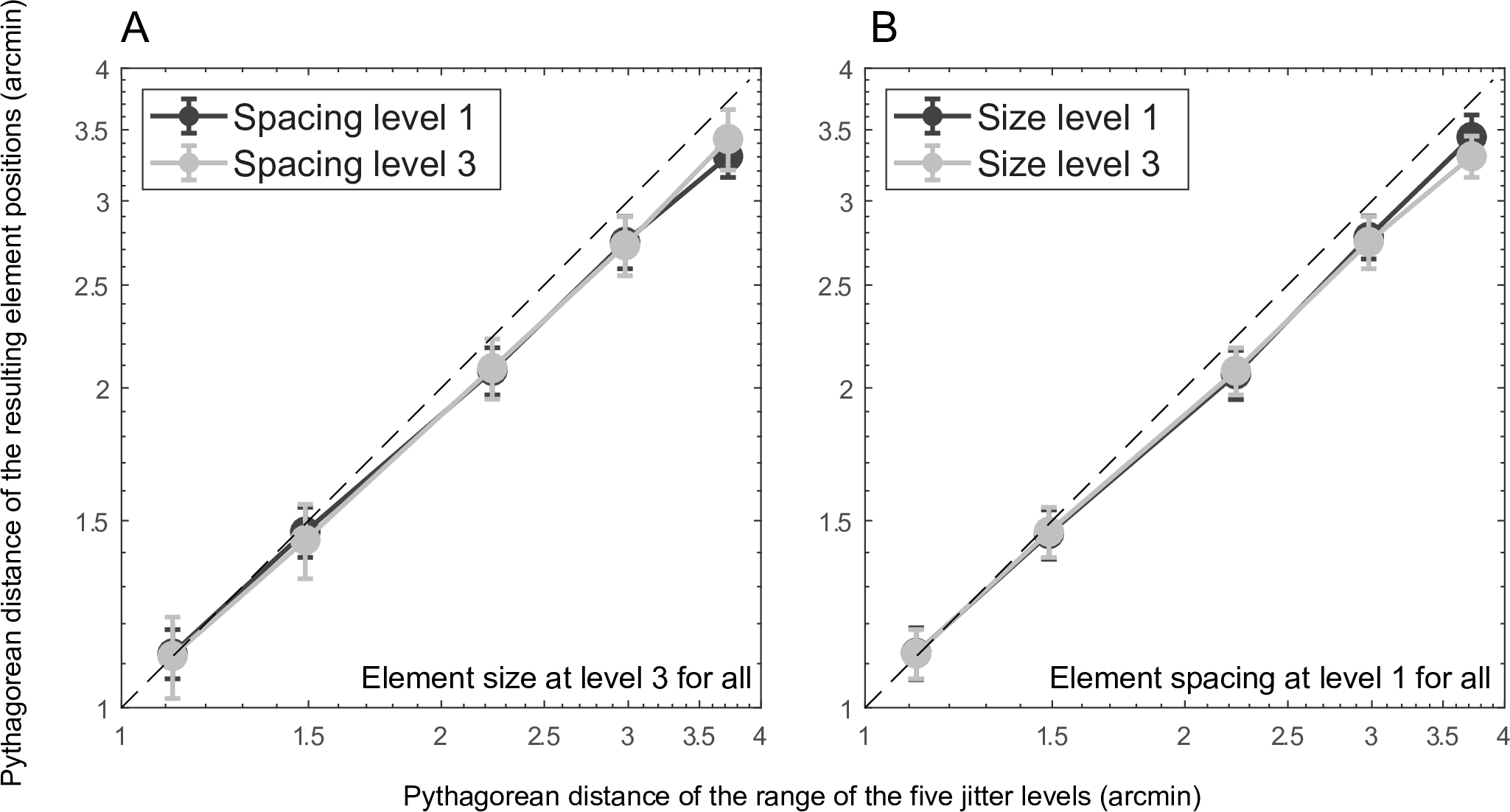

Supplement: S7 Fig — For each data point (which represents one of the total 45 conditions), the actual element positions are calculated from 184 simple images, with a mean jitter value for each image which was averaged across all the element jitter values within that image. The error bars represent the standard deviation (SD) of the 184 mean jitter values. To check whether element spacing and element size influence the final jitter results, we chose the most different conditions to present here. In A, the smallest spacing (dark line) and largest spacing (light line) at the largest element size level show very little difference in the final jitter positions. Similarly in B, the smallest size (dark line) and largest size (light line) at the smallest element spacing level show very little difference in the final jitter positions. All other conditions were also checked with similar results. This analysis demonstrated that the manipulation of jitter is not significantly confounded with the manipulation of element spacing and size. (TIF) [file pcbi.1008802.s007.tif]

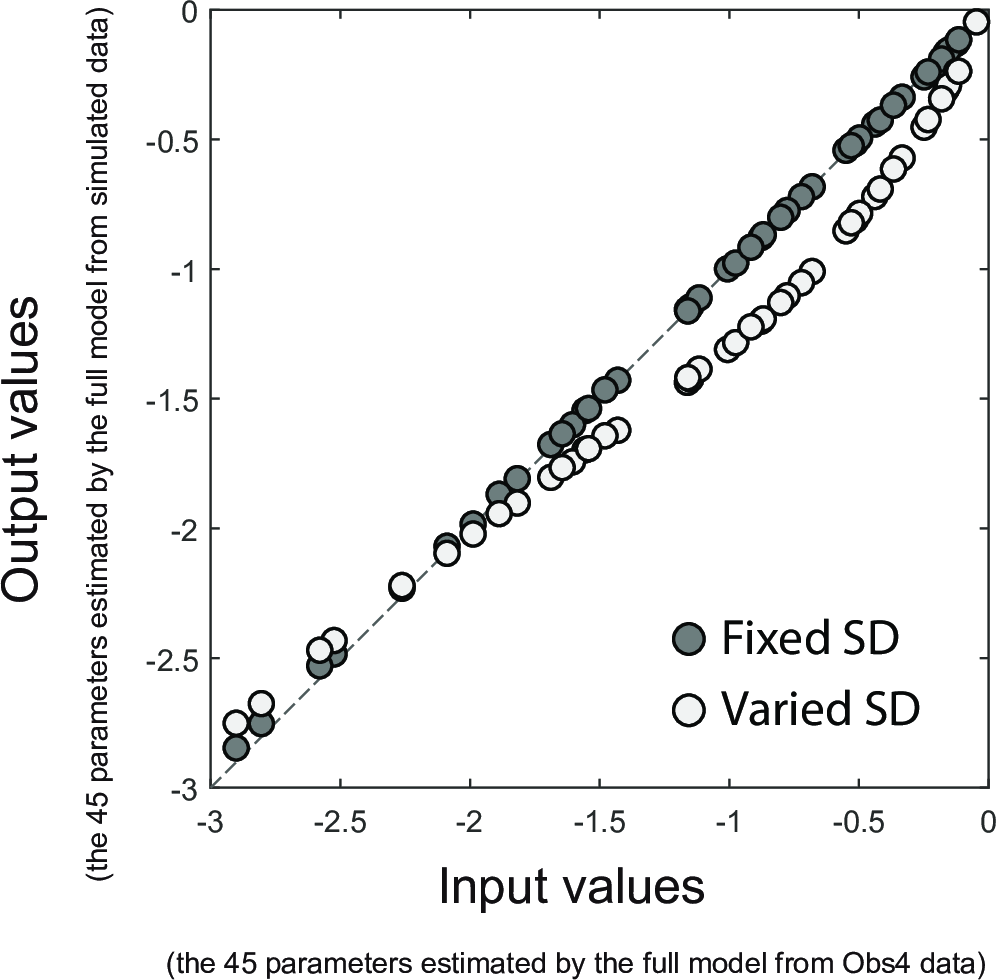

Supplement: S8 Fig — The input values (abscissa) are the 45 parameters in the full model estimated from the real response of a naive participant (Observer 4). The output values (ordinate) are the 45 parameters in the full model estimated from simulated response (1035 pairs × 400 times). To simulate the response, the 45 input parameters were used as the means of normal distributions of the 45 experimental conditions. The standard deviation of each distribution was either from the standard deviation (SD) of the final jitter values as described in S7 Fig, which is varied across the 45 conditions and increase with jitter levels (light dots), or is fixed to the mean SD across the 45 conditions (dark dots). Each of the simulated responses of a pair (e.g. condition A vs. condition B) was then decided by randomly choosing a value from the distribution of condition A and condition B separately, and the larger value receives “more regular” response. For all the possible 1035 pairs, the simulation was repeated for 400 times each, and the total 414,000 simulated responses were then used to estimate the 45 parameters in the full model (ordinate). For fixed SDs for all the 45 distributions (dark dots), the parameter estimation of the full model from real response and simulated response are perfectly matched (the dark dots are aligned with the diagonal), reassuring us that our model-fitting procedure is valid. However, when the SDs of distributions are varied (light dots), the parameter estimation from simulated responses showed a slight deviation from the diagonal. (TIF) [file pcbi.1008802.s008.tif]
