# Supplementary material for: Visual perception of texture regularity: Conjoint measurements and a wavelet response-distribution model
Source: PLoS Comput Biol. 2021 Oct 15;17(10):e1008802. doi: 10.1371/journal.pcbi.1008802 (PMC8550603; doi:10.1371/journal.pcbi.1008802)
Supplement: S1 Table — Deviance values (p-values) for the main effect of element spacing (Model 2), size (Model 3) and jitter (Model 4), tested against the Baseline model (Model 1). The p-values were calculated from likelihood-ratio tests (see Methods for details). (DOCX) [file pcbi.1008802.s009.docx]

|  | Element Spacing | Element Size | Jitter |
| --- | --- | --- | --- |
| Obs 1 | 8.30 (0.016) | 92.66 (<0.001*) | 1723.0 (<0.001*) |
| Obs 2 | 100.95 (<0.001*) | 3.97 (0.14) | 1462.1 (<0.001*) |
| Obs 3 | 116.72 (<0.001*) | 36.23 (<0.001*) | 2232.7 (<0.001*) |
| Obs 4 | 96.52 (<0.001*) | 61.91 (<0.001*) | 1296.7 (<0.001*) |
| Obs 5 | 79.86 (<0.001*) | 15.43 (<0.001*) | 2643.9 (<0.001*) |
| Mean | 80.47 | 42.04 | 1871.68 |

* p-values are significant at Bonferroni-corrected significance level 0.05/5 = 0.01. The degrees of freedom (Df) are 2 for Spacing and Size; 4 for Jitter.
