# Supplementary material for: Visual perception of texture regularity: Conjoint measurements and a wavelet response-distribution model
Source: PLoS Comput Biol. 2021 Oct 15;17(10):e1008802. doi: 10.1371/journal.pcbi.1008802 (PMC8550603; doi:10.1371/journal.pcbi.1008802)
Supplement: S2 Table — Deviance values (p-values) for the interaction effect of element spacing × jitter (Models 5 vs. 8), element size × jitter (Models 6 vs. 9) and element spacing × size (Models 7 vs. 10). (DOCX) [file pcbi.1008802.s010.docx]

|  | Element Spacing × Jitter | Element Size × Jitter | Element Spacing × Size |
| --- | --- | --- | --- |
| Obs 1 | 236.53 (<0.001*) | 93.85 (<0.001*) | 11.12 (0.025) |
| Obs 2 | 117.03 (<0.001*) | 79.39 (<0.001*) | 0.52 (0.97) |
| Obs 3 | 157.79 (<0.001*) | 61.34 (<0.001*) | 1.01 (0.91) |
| Obs 4 | 176.97 (<0.001*) | 44.97 (<0.001*) | 12.80 (0.012) |
| Obs 5 | 200.11 (<0.001*) | 101.48 (<0.001*) | 5.12 (0.28) |
| Mean | 177.69 | 76.21 | 6.11 |

* p-values are significant at Bonferroni-corrected significant level 0.05/5 = 0.01. The Dfs are 8 for spacing × jitter and size × jitter; 4 for spacing × size.
