# Supplementary material for: Visual perception of texture regularity: Conjoint measurements and a wavelet response-distribution model
Source: PLoS Comput Biol. 2021 Oct 15;17(10):e1008802. doi: 10.1371/journal.pcbi.1008802 (PMC8550603; doi:10.1371/journal.pcbi.1008802)
Supplement: S3 Table — Deviance values (p-values) for the three-way interaction effect of element spacing × size × jitter (Models 11 vs. 12). (DOCX) [file pcbi.1008802.s011.docx]

|  | Element Spacing × Size × Jitter |
| --- | --- |
| Obs 1 | 22.11 (0.14) |
| Obs 2 | 25.74 (0.058) |
| Obs 3 | 27.70 (0.034) |
| Obs 4 | 12.95 (0.68) |
| Obs 5 | 46.61 (<0.001*) |
| Mean | 27.02 |

* p-values are significant at Bonferroni-corrected significance level 0.05/5 = 0.01. The Df is 16
