# Supplementary material for: Visual perception of texture regularity: Conjoint measurements and a wavelet response-distribution model
Source: PLoS Comput Biol. 2021 Oct 15;17(10):e1008802. doi: 10.1371/journal.pcbi.1008802 (PMC8550603; doi:10.1371/journal.pcbi.1008802)
Supplement: Attachment — Submitted filename: Rev_PCOMPBIOL-D-21-00121.pdf [file pcbi.1008802.s017.pdf]

Review of ms: PCOMPBIOL-D-21-00121

The authors use maximum likelihood conjoint measurement (MLCM) in a 3-way design to study the effects of spacing, size and jitter on perceived regularity. The results revealed a large influence of jitter on perceived regularity, not surprisingly, and much smaller contributions of spacing and size. Five subjects were tested over time to complete 4 repetitions of the stimulus set and gave broadly consistent results. The authors proceeded to test a series of nested models involving additive and interaction effects of various orders. The results indicated significant 2-way jitter:spacing and jitter:size interactions but neither a significant spacing:size interaction nor a 3-way interaction. Strangely, the 2-way additive vs independence models were not evaluated but given the results for the higher order interactions, there probably isn't a need for these (or for that matter for the independence vs null model tests in Table 1), given the previous results, i.e., if one follows the dictum that marginal effects are not interpretable in the presence of higher order interactions (see sections 5.1-5.2 of https://www.stats.ox.ac.uk/pub/MASS3/Exegeses.pdf). Subsequently, they compared the results to predictions based on the output of a network of Gabor filters distributed over orientation and spatial frequency. The results provided only weak support in favor of a model based on spatial frequency peakedness of the response distribution and instead supported a model in which regularity perception is based on spatial frequency information extracted across orientation channels, as such a model could account for the highest percentage of variance in the data. The experiments and analyses are solid. I have some issues with the terminology and clarity of description of the methods. With these addressed the paper would represent an important contribution to understanding an aspect of texture perception.

1. This is a novel approach in the sense that previous publications using the MLCM technique have studied only 2 dimensions conjointly at a time rather than 3, mostly because of the combinatorial explosion of trials required to make an exhaustive sampling of all pairwise combinations of the attributes. The authors address this problem by using a smaller number of levels along two of the dimensions. A previous study (Gerardin, P, Devinck, F, Dojat, M, Knoblauch, K (2014). Contributions of contour frequency, amplitude, and luminance to the watercolor effect estimated by conjoint measurement. *J Vis*, **14**, 4.) looked at 3 dimensions but only using pairwise comparisons. The authors could have employed this procedure to arrive at similar results for their two-way interactions but would have not been able to test the 3-way interaction. Thus, this is an excellent demonstration of the efficiency of factorial design. Contrary to what the authors claim, however, their approach is not novel because "MLCM methodology was originally designed for two factors..." (p. 27) nor that "Traditional MLCM has provided a framework for handling only two factors..." (p. 20). The theoretical development of the technique in Section 8.2 (pp. 233-236) of the Knoblauch & Maloney book that they reference and also in a more recent review (Maloney, LT, Knoblauch, K (2020). Measuring and Modeling Visual Appearance. *Annu Rev Vis Sci*, **6**:519-537.) is presented in terms of an arbitrary number, N of dimensions, and the OpenSource software R package **MLCM** (https://CRAN.R-project.org/package=MLCM) (Aguillar et al., 2019), has been set-up to accommodate $N \geq 2$ dimensions for at least 10 years (see remark 5, below).

2. Figure 3 is commonly called a Conjoint Proportions Plot, after Ho et al. whom they reference, and it should be designated as such. They elegantly expand it to account for their 3-factor design. As there are only 4 repetitions per stimulus condition, the color values in the plots can only take on 5 different levels. So, I wonder why they use a continuous bar scale coding the proportions that

vary by 10% increments? I find this misleading. In addition, I do not see what is simulated in the plot labelled "Simulated". Normally (and as they describe it), it is calculated simply by assuming that the "ideal" observer chooses the stimulus that is physically greater along one of the scales, here being jitter. No simulation necessary!

3. The authors go to some lengths to spell out the numerous models and contrasts that they employ, but it would be much clearer and more efficiently expressed if they provided an explicit description of the decision rule and how the various nested models relate to it. I found myself having to re-read these sections several times to follow what they were trying to explain. This would make the description of the design matrix on the bottom of p. 26 more lucid. On p. 29, dropping the first levels of the factors is not to minimize the number of parameters but to make the models identifiable. Without such a constraint, there would be no unique fit for the glm. I do not see where they make explicit the assumption about noise in the decision process. Without noise, the observers are expected to make the same response to a repeated stimulus pair. There is some confusion, also, in the naming of the models because of insistence on explaining everything in terms of an ANOVA framework. These are nested models tested with likelihood ratio tests, or more simply nested likelihood ratio tests. ANOVA is like this because it happens to be a special case of a linear predictor of a glm, not the other way around. (See, for example, Prins, N. (2018). Applying the model-comparison approach to test specific research hypotheses in psychophysical research using the Palamedes toolbox. *Frontiers in Psychology*, **9**, 1250).

4. The nested likelihood ratio tests from the models that they fit generate χ^2 statistics. This should be indicated explicitly, i.e., the differences in deviance are described, but nowhere is it indicated how these statistics are distributed or what are the degrees of freedom for the statistics for which the p-values are given.

5. To help me understand the models better (and kudos to the authors for supplying the data), I tried to replicate the likelihood ratio statistics, i.e., the χ^2 values, in the tables, using methods from the **MLCM** package and R, but could only do so partially. I get numbers very close to those of the authors for all subjects except I find that Obs 1 and Obs 2 deviate some. If my results were off for all subjects, I might think that the methods that I employed were incorrect but since I can reproduce the values for some of the subjects, I wonder if there are errors of transcription in the tables. These should be checked, in any case. My R code and results are given below, assuming that the data files that they supplied as Supplementary data are in the working directory.

```
library(MLCM)

dr <- dir(pattern = ".csv")

# S spacing, Z size, J jitter
d.lst <- lapply(dr, function(f){
    d <- read.csv(f, header = FALSE)
    names(d) <- c("Resp", paste0(rep(c("S", "Z", "J"), each = 2), 1:2))
    as.mlcm.df(d)
})
```

Table 1 χ^2 values

```
#  Table 1:  independence vs null model
T1 <- cbind(
Spacing = sapply(d.lst, function(d){
    sm <- summary(mlcm(d, model = "ind", whichdim = 1)$obj)
```

```r
    round(sm$null.deviance - sm$deviance, 2)
}),

Size = sapply(d.lst, function(d){
    sm <- summary(mlcm(d, model = "ind", whichdim = 2)$obj)
    round(sm$null.deviance - sm$deviance, 2)
}),

Jitter = sapply(d.lst, function(d){
    sm <- summary(mlcm(d, model = "ind", whichdim = 3)$obj)
    round(sm$null.deviance - sm$deviance, 2)
})
)
rownames(T1) <- paste("Obs", 1:5)
T1
```

```
##       Spacing   Size  Jitter
## Obs 1   20.64 135.56 1745.75
## Obs 2  100.95   3.97 1462.11
## Obs 3  116.71  36.23 2232.66
## Obs 4   96.52  61.91 1296.74
## Obs 5   79.86  15.43 2643.93
```

Table 2 χ^2 values

```r
# Table 2:  2 way interaction vs 2 way additive
T2 <- cbind(
SpacexJitter = sapply(d.lst, function(d){
    round(mlcm(d[, -c(4, 5)])$obj$deviance -
        mlcm(d[, -c(4, 5)], model = "full")$obj$deviance, 2)
}),

SizexJitter = sapply(d.lst, function(d){
    round(mlcm(d[, -c(2, 3)])$obj$deviance -
        mlcm(d[, -c(2, 3)], model = "full")$obj$deviance, 2)
}),

SpacexSize = sapply(d.lst, function(d){
    round(mlcm(d[, -c(6, 7)])$obj$deviance -
        mlcm(d[, -c(6, 7)], model = "full")$obj$deviance, 2)
})
)
rownames(T2) <- paste("Obs", 1:5)
T2
```

```
##       SpacexJitter SizexJitter SpacexSize
## Obs 1       174.85      128.71      12.94
## Obs 2       117.03       79.39       0.52
## Obs 3       157.79       61.34       1.01
## Obs 4       176.97       44.97      12.80
```

```
## Obs 5       200.11      101.48       5.12
```

Table 3 χ^2 values

```r
#  Table 3: 3-way interaction tested against 3 two-way interactions
SizexSpacexJitter <- sapply(1:5, function(ix){
    m1 <- mlcm(d.lst[[ix]], model = "full")$obj$deviance
    mm <- cbind(model.frame(mlcm(d.lst[[ix]][, -c(4, 5)], model = "full")$obj),
        model.matrix(mlcm(d.lst[[ix]][, -c(2, 3)], model = "full")$obj),
        model.matrix(mlcm(d.lst[[ix]][, -c(6, 7)], model = "full")$obj)
    )
    g <- glm(Resp ~ . + 0, family = binomial(probit), data = mm)$deviance
    g - m1
})
names(SizexSpacexJitter) <- paste("Obs", 1:5)
cbind(SizexSpacexJitter)
```

```
##       SizexSpacexJitter
## Obs 1          31.83050
## Obs 2          25.74348
## Obs 3          27.69612
## Obs 4          12.94537
## Obs 5          46.61027
```

6. On p. 16, Why simulate from the full model, model 12, when the 3-way and 2-way space:size interactions were not significant?
7. In the abstract, the abbreviation SF is introduced for spatial frequency before it is defined.
8. p. 19, The so-called ANOVA approach (it is not variance but deviance which is being analyzed) is not novel. It is standard in testing of nested models that was first outlined for MLCM in the Ho et al. paper when defining the series of nested models: independence, additive and full. With a 3-factor design, the possibilities are expanded, as they have done, in a fashion that is standard for glm's, which provide the underlying framework for performing the maximum likelihood fits here.
9. It's generally considered misleading to make bar charts that do not start from a zero baseline (e.g., https://thenode.biologists.com/non-zero-baselines-the-good-the-bad-and-the-ugly/resources/). Of course, this isn't possible with log ordinates, as in Figure 7. I wonder if box-and-whisker plots might not work better, with the individual data points still added as in the current figure?
