# Supplementary material for: Visual perception of texture regularity: Conjoint measurements and a wavelet response-distribution model
Source: PLoS Comput Biol. 2021 Oct 15;17(10):e1008802. doi: 10.1371/journal.pcbi.1008802 (PMC8550603; doi:10.1371/journal.pcbi.1008802)
Supplement: Attachment — Submitted filename: response letter R2_final.pdf [file pcbi.1008802.s018.pdf]

Reviewer 2 comments:

The authors use maximum likelihood conjoint measurement (MLCM) in a 3-way design to study the effects of spacing, size and jitter on perceived regularity. The results revealed a large influence of jitter on perceived regularity, not surprisingly, and much smaller contributions of spacing and size. Five subjects were tested over time to complete 4 repetitions of the stimulus set and gave broadly consistent results. The authors proceeded to test a series of nested models involving additive and interaction effects of various orders. The results indicated significant 2-way jitter:spacing and jitter:size interactions but neither a significant spacing:size interaction nor a 3-way interaction. Strangely, the 2-way additive vs independence models were not evaluated but given the results for the higher order interactions, there probably isn't a need for these (or for that matter for the independence vs null model tests in Table 1), given the previous results, i.e., if one follows the dictum that marginal effects are not interpretable in the presence of higher order interactions (see sections 5.1-5.2 of https://www.stats.ox.ac.uk/pub/MASS3/Exegeses.pdf).Subsequently, they compared the results to predictions based on the output of a network of Gabor filters distributed over orientation and spatial frequency. The results provided only weak support in favor of a model based on spatial frequency peakedness of the response distribution and instead supported a model inwhich regularity perception is based on spatial frequency information extracted across orientation channels, as such a model could account for the highest percentage of variance in the data. The experiments and analyses are solid. I have some issues with the terminology and clarity of description of the methods. With these addressed the paper would represent an important contribution to understanding an aspect of texture perception.

It is not quite right to say that the SF × Orientation distribution model enjoys only weak support, since all the evidence shows superior performance of this model compared to the others. As predicted by the SF × Orientation model, the energy concentrations (Fig. 9) were affected much more by jitter than by element spacing or size, in line with our behavioural data (Fig. 7). Moreover this model works for natural images too (Supplementary Fig. 6). Importantly, the variance of SF-distribution skew across orientations in the SF × Orientation distribution model can explain 70% of the variance of behavioral data, which is generally considered strong.

We agree that marginal effects should be interpreted with caution when higher-order interactions are significant. However, we also think the marginal effects might provide additional information and be of interest to many readers. We have now added a comment on page 8 in the revised manuscript about this issue.

1. This is a novel approach in the sense that previous publications using the MLCM technique have studied only 2 dimensions conjointly at a time rather than 3, mostly because of the combinatorial explosion of trials required to make an exhaustive sampling of all pairwise combinations of the attributes. The authors address this problem by using a smaller number of levels along two of the dimensions. A previous study (Gerardin, P, Devinck, F, Dojat, M, Knoblauch, K (2014). Contributions of contour frequency, amplitude, and luminance to the watercolor effect estimated by conjoint measurement. *J Vis*, **14**, 4.) looked at 3 dimensions but only using pairwise comparisons. The authors could have employed this procedure to arrive at similar results for their two-way interactions but would have not been able to test the 3-way interaction. Thus, this is an excellent demonstration of the efficiency of factorial design. Contrary to what the authors claim, however, their approach is not novel because "MLCM methodology was originally designed for two factors. . . " (p. 27) nor that "Traditional MLCM has provided a framework for handling only two factors. . . " (p. 20). The theoretical

development of the technique in Section 8.2 (pp. 233-236) of the Knoblauch& Maloney book that they reference and also in a more recent review (Maloney, LT, Knoblauch, K (2020). Measuring and Modeling Visual Appearance. *Annu Rev Vis Sci*, **6**:519-537.) is presented in terms of an arbitrary number, N of dimensions, and the OpenSource software R package **MLCM** (https://CRAN.R-project.org/package=MLCM) (Aguillar et al., 2019), has been set-up to accommodate $N \geq 2$ dimensions for at least 10 years (see remark 5, below).

Thank you for pointing out these important papers. However the study by Gerardin et al. (2014) is not a real 3-dimensional MLCM; it is essentially a 2-dimensional MLCM run separately on pairwise combinations of 3 factors (A×B, B×C and A×C). For a 5×5×5, 3-dimensional MLCM, there should be 7750 possible pairs; but for three of 5×5, 2-dimensional MLCM (as in Gerardin et al., 2014) there are only 300×3 = 900 possible pairs. Therefore our implementation of MLCM is quite different from that of Gerardin et al. (2014) and we maintain that we are the first to perform a fully 3-dimensional MLCM on behavioral data.

It is true that as described by Maloney & Knoblauch, MLCM can accommodate data with more than 2 dimensions (and thank you for pointing out the need to correct the relevant statement in our manuscript on page 21). However, if we had fitted the conventional 3-dimensional MLCM to our data, we would have been unable to analyze how the difference in performance between the additive and full models is distributed across 2-way and 3-way interactions. A solution to this problem, as you mention, is to fit the 3 pairwise combinations of the 3-factor MLCM (Gerardin et al., 2014), but had we done this we would have been unable to test for a possible 3-way interaction. An inability to test for a 3-way interaction would have been problematic, since 2-way interactions are hard to interpret in the presence of a 3-way interaction. Considering 2-way interactions without checking for 3-way interactions could lead to misleading conclusions, since the 2-way interactions may only be important at specific levels of the third factor. Our approach allows us to split the deviance explained by the full model into 1-way, 2-way and 3-way effects. We believe this is an important contribution to the existing MLCM technique. Please see our changes to page 10, 20, 21

2. Figure 3 is commonly called a Conjoint Proportions Plot, after Ho et al. whom they reference, andit should be designated as such. They elegantly expand it to account for their 3-factor design. As there are only 4 repetitions per stimulus condition, the color values in the plots can only take on5 different levels. So, I wonder why they use a continuous bar scale coding the proportions that vary by 10% increments? I find this misleading. In addition, I do not see what is simulated in the plot labelled "Simulated". Normally (and as they describe it), it is calculated simply by assumingthat the "ideal" observer chooses the stimulus that is physically greater along one of the scales, here being jitter. No simulation necessary!

Thanks for the suggestions. In the revision, we now refer to this as a Conjoint Proportions Plot in the revised text (page 7) and Legend for Figure 3 (page 37), as well as Figure 3 itself. We have also now changed "Response matrix" and "Simulated" to "Conjoint Proportions Plot" and "ideal", respectively, in Figure 3.

It is true that the color values in the individual plots can only take 5 different levels (0%, 25%, 50%, 75% and 100%). However the group mean values can take many more intermediate levels so we need to use a continuous scale here. This is now clarified in the revised manuscript, on page 7.

3. The authors go to some lengths to spell out the numerous models and contrasts that they employ, but it would be much clearer and more efficiently expressed if they provided an explicit description of the decision rule and how the various nested models relate to it. I found myself having to re-read these sections several times to follow what they were trying to explain. This would make the description of the design matrix on the bottom of p. 26 more lucid. On p. 29, dropping the first levels of the factors is not to minimize the number of parameters but to make the models identifiable. Withoutsuch a constraint, there would be no unique fit for the glm. I do not see where they make explicit the assumption about noise in the decision process. Without noise, the observers are expected to make the same response to a repeated stimulus pair. There is some confusion, also, in the naming of the models because of insistence on explaining everything in terms of an ANOVA framework. These are nested models tested with likelihood ratio tests, or more simply nested likelihood ratio tests. ANOVA is like this because it happens to be a special case of a linear predictor of a glm, not the other way around. (See, for example, Prins, N. (2018). Applying the model-comparison approach to test specific research hypotheses in psychophysical research using the Palamedes toolbox. *Frontiersin Psychology*, **9**, 1250).

   We do provide an explicit – although brief - description of the decision rule prior to describing the different models. We have now made this explanation more detailed and hopefully easier to understand in the Data Analysis section of the Methods (page 30).

   Indeed, the reason to drop the first level of factors is to make the model fits unique, which is what we meant by avoiding redundancy. We now realize this is confusing and have changed the manuscript accordingly (page 32).

   We have now included internal noise in the description of the models, on page 30 in the revised manuscript.

   The statistical assumptions behind ANOVAs and nested likelihood ratio tests are fundamentally different, and we understand that referring to our procedure as an 'ANOVA approach' can be confusing to the reader. We have accordingly changed "ANOVA-based statistical models" to "Three-way MLCM statistical models" throughout the revised manuscript. We have also made it clearer that we use nested likelihood ratio tests (page 30), and refer to "nested models" on pages 9 and 11)

4. The nested likelihood ratio tests from the models that they fit generate χ^2 statistics. This should be indicated explicitly, i.e., the differences in deviance are described, but nowhere is it indicated howthese statistics are distributed or what are the degrees of freedom for the statistics for which the p-values are given.

   Thanks for reminding us. We have now specified the χ^2 distribution in the likelihood ratio tests in the Data Analysis section (page 30), and the degrees of freedom are now added to the descriptions of Tables 1-3.

5. To help me understand the models better (and kudos to the authors for supplying the data), I tried to replicate the likelihood ratio statistics, i.e., the χ^2 values, in the tables, using methods from the **MLCM** package and R, but could only do so partially. I get numbers very close to those of the authors for all subjects except I find that Obs 1 and Obs 2 deviate some. If my results were off forall subjects, I might

think that the methods that I employed were incorrect but since I can reproduce the values for some of the subjects, I wonder if there are errors of transcription in the tables. Theseshould be checked, in any case. My R code and results are given below, assuming that the data files that they supplied as Supplementary data are in the working directory.

Thank you for trying to replicate our results. We have found three reasons why our results slightly differ:

1. We unfortunately uploaded the wrong file for Obs 1. That was the first author's pilot data on another version of the experiment (Exp 358). We apologize for this mistake; the correct set of experimental data (Exp 359) is now re-uploaded.
2. Our original models include an intercept value while yours does not. In retrospect we realize this analysis is a very special case where it is more appropriate to not estimate an intercept (as done by Ho et al). We have now removed the intercept in our models (pages 31 to 34), and recalculated the results accordingly.

By taking these matters into account, we are able to replicate the results you've sent us perfectly.

6. On p. 16, Why simulate from the full model, model 12, when the 3-way and 2-way space-size interactions were not significant?

   We simulate from the full model because we want the best estimate of regularity of each image for the SF x Orientation distribution section. In this specific scenario, we think it is better to choose the model that explains the most deviance rather than worry about type II errors. Since we have a large number of trials for each image, we think introducing 16 more parameters will lead to better estimates without much overfitting. In other words, with the amount of data we have for each participant, we think it is better to reduce the bias rather than the variance of the model estimates.

   In addition, the 3-way interaction is significant for one of the participants (#5), and the effect is moderate for participants 2 and 3.

   We have now explained this in the Results (page 17).

7. In the abstract, the abbreviation SF is introduced for spatial frequency before it is defined.

   Revised. Thanks for the reminder.

8. p. 19, The so-called ANOVA approach (it is not variance but deviance which is being analyzed) isnot novel. It is standard in testing of nested models that was first outlined for MLCM in the Ho etal. paper when defining the series of nested models: independence, additive and full. With a 3-factor design, the possibilities are expanded, as they have done, in a fashion that is standard for glm's, which provide the underlying framework for performing the maximum likelihood fits here.

Thanks for pointing this out. We now have made this sentence less ambiguous (page 20). Please also see our response in question 3.

9. It's generally considered misleading to make bar charts that do not start from a zero baseline (e.g., https://thenode.biologists.com/non-zero-baselines-the-good-the-bad-and-the-ugly/resources/). Of course, this isn't possible with log ordinates, as in Figure 7. I wonder if box-and-whisker plots might not work better, with the individual data points still added as in the current figure?

   Thanks for the suggestion. We now changed Fig 7 to a linear bar graph starting from 0 and add a break between 250-1250 to improve the visualization of small values.
